# Putative juvenile terrorists: the relationship between multiple traumatization, mental health, and expectations for reintegration among Islamic State recruited adolescent and young adult fighters

**DOI:** 10.1186/s13031-022-00489-3

**Published:** 2022-11-10

**Authors:** Rezhna Mohammed, Frank Neuner

**Affiliations:** 1 Department of Psychology, Clinical Psychology and Psychotherapy, Bielefeld University, Bielefeld, Germany; 2 Vivo International, Constance, Germany; 3 Institute for Interdisciplinary Research on Conflict and Violence, Bielefeld University, Bielefeld, Germany

**Keywords:** Reintegration, Mental health, Trauma exposure, Child soldiers

## Abstract

**Background:**

In several conflicts worldwide children are recruited as fighters in irregular forces. These children need to be reintegrated into the society after the conflict. However, concurrent to various reservations in the communities, the reintegration of former child soldiers is challenged by the fact that many of the affected children were indoctrinated by the armed group and traumatized through war events. Even several years after the defeat of the terrorist organization ISIS in Iraq, systematic efforts towards the reintegration of children who had been recruited by ISIS are notably absent*.*

**Methods:**

we conducted clinical interviews with a sample of N = 59 adolescents and young adults who were incarcerated for terrorism in the prisons of the Kurdistan Region of Iraq to assess levels and types of trauma exposure, PTSD, depression, readiness to reintegrate and ongoing identification with ISIS.

**Results:**

We found high levels of PTSD and depression that were associated with trauma exposure. The subjective readiness to reintegrate into the communities was associated with trauma exposure and was mediated by depression, even after controlling for the influence of ongoing identification with the armed group.

**Conclusion:**

The study indicates that trauma-related mental ill-health should be considered in efforts to reintegrate young former terrorists.

## Background

Children are among the most vulnerable groups during war and armed conflict, and they often fall victim to various grave violations, such as recruitment and use as soldiers by both legal and illegal forces including armed and terrorist groups. The United Nations Security Council has identified and condemned 6 grave violations against children during war and conflict among which recruitment in armed groups and armed forces is mentioned. In the period between 2005 and 2020 UNICEF verified more than 93,000 children to have been recruited and used by armed groups, with the actual number being much higher than what is reported [75, 78]

### The mental health outcome of child soldiering

A range of studies from African countries shows that child soldiers have high rates of posttraumatic stress disorder (PTSD), depression, anxiety, emotional regulation problems, and externalizing behaviors [22, 29, 30, 63].

Child soldiers are among the most vulnerable victims of current conflicts, presenting with a wide range of devastating experiences during critical periods of their development. Warfare increasingly targets the civil population and brings about high rates of adversities and related psychological disorders [6, 15, 16, 49, 50, 53].

### A threatening environment and its impacts on the mental health of the child

In recent years, more and more studies are looking into the social ecology of child soldiering [5, 39, 40, 41] the social-ecological theory that was first introduced by Bronfenbrenner in 1979 looks at social relationships as determinants of an individual's behavior and well-being [11]. It looks at the immediate environment of the individual such as the family (the microsystem), and the exosystem, how the neighborhood and social support groups impact the behavior and well-being of the individuals and families, and the macrosystem which is represented in the institutions that shape or constrain the behavior such as policies and economic environment [17].

In addition to the direct effect of war and conflict on children [38], there are also multiple indirect by-products of war and conflict that results in creating a dangerous environment for children (both on the exosystem and the macrosystem), particularly in domestic settings. For instance, war and conflict increase the rates of intimate partner violence [25, 34] as well as familial maltreatment and child abuse [14, 15, 15, 16, 16, 61, 65]. On top of this highly threatening environment, child soldiers also experience violence within their organization and direct threats in combat [4].

Experiencing this accumulation of potentially traumatic events can have a lasting and damaging impact on the growing mind and body of the child [20, 62]. Childhood adversity is linked to impaired physical [54, 72] and mental health [18, 46, 52] that extends beyond childhood. The number of *types* of traumatic events is a key predictor of psychological disorders [21, 27, 58]. Children and adolescents who encounter several types of childhood adversity experiences have more mental health problems [2, 10] such as depression [8, 24], PTSD [10, 66], and attention and behavioral problems [35, 60, 79] that often remain chronic throughout life [43, 72]. The effect of polyvictimization, the simultaneous exposure to multiple types of trauma during childhood, is linked to PTSD, depression, and anxiety [27], as well as delinquency [57] and, is also evident in war-affected children [14]. Within this population, adolescents and young adults who are imprisoned due to terrorist activities after a conflict may be especially vulnerable since juvenile detention is independently associated with poor health outcomes [44].

### Terrorism as an added layer of threat to the child

Terrorism comes with added layers of vulnerability for children and youth. The security response to terrorism raises protection challenges for children. Children who are stuck in the middle of counter-terrorism operations are often killed or maimed. Those who survive are usually arrested and detained for being allegedly involved with armed and terrorist groups. Children who end-up in the justice system are often treated as threats rather than victims. Many are arrested and detained, often for extended periods of time and without due process, with the risk of facing further violations of their rights such as torture, physical and sexual violence, and deprivation of access to their basic needs, and health and education services. Some of these children are even tried in military courts [76]

### Reintegration of child soldiers

After the conflict, although some child soldiers may be placed in rehabilitation centers, most often they are imprisoned. Proper rehabilitation and reintegration of child soldiers into civil society is critically important to the functioning of both these individuals and their wider communities. To achieve this, the experiences of this group of children must be considered.

Effective reintegration of child soldiers is a complex and long-term undertaking. It is multiyear and multisectoral with mental health and psychosocial support as a key components for effective reintegration. Despite the obvious importance of proper reintegration, there is still a long way before it reaches consensus, especially when it comes to how to measure it and what works best [7, 37], this is understandable given the complex nature of reintegration, as put by Kohrt et al. [41], for reintegration programs to work, it needs to be situated in the human biology and anthropology sciences, he argues that in order to successfully reintegrate child soldiers, we need to consider the direct social environment in which the child is to return to, and at that, reintegration programs must vary from a country to another but also from a community to another [41] Additionally, reintegration programs can take years to achieve its goals, making it one of the most expensive programs, in their report, the global coalition for reintegration of child soldiers calls for establishing a global monitoring framework for reintegration, they also argue that for reintegration programs to be effective, it needs to be planned for a minimum of 3–5 years [77].

### The context of Iraq

After some decline in terrorist activities in 2014, Iraq has remained among one of the most affected countries by terrorism and activities of irregular forces [12, 74]. Alongside ISIS, there are several other active terrorist groups in Iraq, operating against the background of ongoing conflicts between the different ethnic and religious groups in the country [67, 81]. To date, research on former children employed in irregular forces has been focused on the African context. However, other regions, particularly in the Middle East in countries like Syria and Iraq, have been affected by armed conflicts where terrorist forces have occupied multiple towns and regions for extended periods of time.

### The current study

This study had the unique opportunity to access a sample of adolescents and young adults who were incarcerated for terrorism-related crimes and were living in the juvenile facilities of the Kurdistan Region of Iraq (KRI). We developed scales to assess their subjective readiness to be reintegrated into the communities as the outcome variable since we assumed that one prerequisite for a successful reintegration would be positive expectations toward life in their communities of origin. In addition, we developed an instrument to measure ongoing identification with ISIS, to index this important factor that may undermine one’s readiness for reintegration. Considering that impaired mental health, in particular high levels of depression, have been shown to interfere with positive expectations [73], we hypothesized that readiness for reintegration would be negatively correlated with depression and ongoing identification with ISIS.

## Method

### Participants

The sample was recruited from residents of a juvenile prison in Erbil, Kurdistan Region of Iraq (KRI). Purposive sampling was used to select the study participants. We selected participants who were convicted of terrorism crimes under Iraqi law and invited them to be interviewed. All participants were male (*N* = 59). The mean age was 18 years (SD = 2.29, range: 14–24). The vast majority of the sample were adolescents during their membership with ISIS. Most of the participants were Arabs (93.2%), of Sunni Muslim religion (84.7%), 5.1% were Kurds and 1.7% were Turkmans, they were from different towns, cities, and villages in the center and north of Iraq. History of displacement was high among the sample, with 79.7% having been displaced from their city of origin at least once. Overall, 28.8% were married, and 69.5% were single. The average years of formal schooling were M = 7 years (SD = 2.50). A high percentage of the sample, 89.8%, met the criteria for depression, and 69.5% met the criteria for PTSD. Even though prisoners were convicted of ISIS-related crimes, only 78% of the sample reported having joined ISIS or another terrorist group. Experiences of torture during imprisonment were high, endorsed by 83.1% of the sample, for more information on the sample, please refer to Table 1.

**Table 1:** Sociodemographic characteristics of the sample

		M	Range	Count	N %
Age		18 (2.29)	14–24 years		
Ethnicity	Arab			55	93.2
	Kurd			3	5.1
	Turkman			1	1.7
Religion	Muslim Shia			9	15.3
	Muslim Suni			50	84.7
Mother Tongue	Arabic			55	93.2
	Kurdish			3	5.1
	Turkmanish			1	1.7
Marital Status	Single			41	69.5.0
	Married			17	28.8
	Separated			1	1.7
Do you have children	No			44	74.6
	Yes			15	25.4
Years of formal education		7 (2.50)	0–11		
Days in prison		520 (283)	92–1096 days		
Did you join ISIS?	No			13	22.0
	Yes			46	78.0
Is your mother alive?	No			48	81.4
	Yes			11	18.6
Is your father alive?	No			46	78.0
	Yes			13	22.0
Number of Siblings	(3–5)			27	45.8
	(6–8)			22	28.8
	(9 +)			10	17.0
Have you been displaced?	No			12	20.3
	Yes			47	79.7
Have you been tortured?	No			51	86.4
	Yes			8	13.6

### Procedure

This study was carried out with the permission of the General Directory of Social Reformatory in the Kurdistan Region of Iraq (KRI) between 2018 and 2021. This directory manages all prisons in the region. After extended meetings with the staff at the prison, a seminar with the inmates was conducted by the first author to explain the goals and procedures of the study and to give the prisoners an opportunity to choose to participate in the study. The purpose of the study was made fully transparent to the inmates during the seminar. After that, face-to-face interviews began with the study sample. A list of the inmates was initially provided by the department of social workers at the prison to the first author. The first author accompanied a guard to the cells of the inmates to invite them to the interview, returned with the inmate to the interview room and started the interview with an introduction, and detailed informed consent was read to the participants at the beginning of the one-on-one interview. The prison management agreed to allow the first author to bring the printed interview questions and a pen in a backpack into the interview room, at the end of each interview, the first author put the answered forms into the backpack, at the end of the interview day, the first author carried the forms in the backpack outside and took them home, no one of the prison staff have ever requested to see the forms, neither the blank nor the filled ones. All participants who were approached agreed to the interview. All interviews took place in the Iraqi dialect of the Arabic language and were conducted in an office that was dedicated to the interviewer for the period of the interviews, inside the women and juvenile reformatory prison in KRI. The ethical committee of Bielefeld University reviewed the study and approved it (reference number: EUB 2018 – 093), for the full ethical approval statement see the appendix.

### Protection, safety and security

To protect the confidentiality of the participants in the prison setting, all interviews took place in a private room away from the management offices and on the same floor as the prison cells. Only the researcher and the participant were in the room during the interview. As part of the study information, participants were informed about the presence of an international organization that provides mental health and psychosocial support services inside the prison, this NGO had two trained psychotherapists and a psychiatrist. The psychiatrist visited once a week and on an on-need-basis. A referral system with this NGO was initiated by the main researcher before the interviews took place. If requested by the participant, referral was made by the researcher directly to the NGO, during which the first author contacted the staff member of the NGO directly and gave a brief overview of the problem and arranged a date and time for the participant to be seen by the staff member of the NGO, the date and time was later shared with the participant by the first author. Participants were made aware that as part of the referral meeting some initial information would be shared with the psychotherapist/psychiatrist, such as suicidality and the severity of mental health symptoms as shown in the assessment answered by the participant. Periodic follow-up throughout the interviewing period were made by the first author via meetings with the psychotherapist/psychiatrist, if such requested was made by the participant prior to the referral.

### Instruments

Some study instruments were developed for the purpose of this study, and others were adapted following an extensive preparation phase. To ensure an informed face-validity of the instruments, in-depth interviews were carried out with key informants within the United Nations and other humanitarian organizations working with the study sample as well as with government officials in the directory of reformatories and within the prisons. In addition, focus group discussions were conducted with selected participants from the study sample in the prisons. During the focus group discussions, we explored the prisoners' perspectives on ideologies, traumatic experiences, and mental health as well as issues regarding confidentiality and openness to disclosure. One outcome of these focus groups was the development of a list of possible ISIS-related events and perpetration events to be used subsequent interviews. The package of study interview scales was then discussed in further focus groups to ensure its feasibility for the study sample. Some of the scales (demographics, perpetration events, identification with the terrorist group, reintegration items and war related events) were developed in Arabic languages for the current study, while Arabic translations of the scales used to assess PTSD, depression and trauma exposure (PCL-5, D-HSCL and WAEC) were already available. Finally, the family violence scale was translated into Arabic using established translation procedures that involved a blind back-translation and a discussion of the discrepancies between original and back-translation involving both, experts as well as participants. All instruments were administered in an interview format, where participants were first asked demographic questions followed by an administration of the history of violence and the mental health scales.

#### Demographics

Demographic questions included those relating to age, marital status, number of children, ethnicity, religion, and education were asked. Additionally, participants were asked questions regarding imprisonment, displacement, and whether or not they have joined armed groups.

#### Family violence

Exposure to violence was assessed by focusing on different time periods of the ex-combatants life, starting with childhood. A modified version of the family violence scale [15, 16] was administered. Participants were asked to reflect on their childhood and were asked about different types of physical (e.g., “have you been hit?”), verbal (“have you been told you are no good?”) and sexual violence (“have you been raped?”), as well as experiences of neglect (“have you been neglected?") that were perpetrated by a caregiver. All questions were dichotomous and could be answered by yes or no. A total score that summed any instance of experienced and/or witnessed family violence was used in the present study.

#### ISIS events

During the focus groups, it became apparent that some traumatic experiences were unique to ISIS. Accordingly, we created an 8-item scale to capture these experiences. Each item was reported during the focus group discussions and corroborated by experts and service providers on the ground. The scale was created in Arabic and was shown to a native Arabic-speaking psychologist for review, after minor modifications by this psychologist, it was also shown to another psychologist to go over it, a final version of the instrument was created. This final version was then tested in a group with 15 individuals of the study participants to test the language feasibility, the version was easy to understand by the group and was considered a final version that was later used in the individual interviews with the study sample. Participants were asked to think about the period in which they lived under ISIS’s rule before answering the questions. Examples of items include: “Have you witnessed the mass punishments done by ISIS against civilians such as beheading, flogging, etc.  ” and “ Have you been imprisoned by ISIS –  ”. Participants provided “yes” or “no” responses, and affirmative responses were summed to create a total score. The full scale is provided in the supplemental material of this article.

#### War events

Exposure to war events was determined using the War and Adversity Exposure Checklist (WAEC), a validated Arabic instrument created for the use in Iraq [36]. WAEC is a 29-item scale created based on previous studies with Iraqi internally displaced persons in the camps of the Kurdistan Region of Iraq. This scale assesses common traumatic experiences and adversities in war regions, including food and water deprivation, loss of loved ones due to war, and exposure to events such as armed combat and executions. Participants were asked whether they have ever experienced those events and were requested to provide a “yes” or “no” response and a total score was calculated by adding the “yes” answers.

#### Trauma exposure

In order to examine the cumulative *trauma exposure* experienced by the sample over the lifespan, we created the composite variable *trauma exposur*e from the scales assessing family violence, war events, and ISIS-related events. The scale was created as the mean of the standardized z-scores of each variable. The scale’s internal consistency was α = 0.869.

#### Perpetration events

Perpetration events were assessed by presenting eight types of violence that were associated with ISIS and other armed groups in the region. The scale was developed for the purpose of this study. The questions were developed through the focus group discussions with the study sample and were additionally informed by the research team’s field experience. After creating the initial instrument in Arabic, a native Arabic-speaking psychologist reviewed the questionnaire and made linguistic changes, this version was used in a group interview with 15 individuals of the study sample to test its lingual feasibility, the participants found the questionnaire to be well written and easy to understand, after this interview the measure was considered finalized and was used in the individual interviews with the study sample. Participants were prompted to think about the period in which they were with ISIS before answering the questions. For each item, participants were asked to provide “yes” or “no” responses. Items included questions like, “have you attacked another armed group?—  ” and “did you torture anyone?—  ”. “Yes” responses were summed to create a total score of different types of perpetration events. The full scale is available in the supplemental materials. The reliability of the scale was acceptable, α = 0.68. The full scale is provided in the supplemental material of this article.

#### Identification with ISIS

We developed a novel scale tailored specifically to measure participants’ identification with the terrorist group ISIS. This scale was constructed as a result of the focus group discussions with prisoners and key informants within UN Agencies and Humanitarian Organizations. Afterward, relying on the social identity theory and the literature on group identity and group membership (), the instrument was created in Arabic, the first draft was then reviewed by a native Arabic-speaking psychologist who made necessary changes and modified the language accordingly, and another native Arabic psychologist reviewed the language and provided inputs, both versions were then compiled by the first author and changes were integrated. A group interview was held by the first author to test the language accessibility and the overall feasibility of the instrument with 15 participants of the study sample, finally, a version of 18 items was made to be used in the individual interviews with the study sample. Each of item represents a central belief associated with Islamist extremists, an attitude towards the group, or a behavior compatible with the group. For example, the following statements were included: “participation in the fight with ISIS is a moral obligation—  ”, “some of the goals of the group are right for my ethnic/religious group—  ”, “I had friends within the group that I miss now—  ” and “I appreciate the skills I learned within the group—  . Before answering the questions, the participants were asked to answer whether the items represented their beliefs, attitude, and behavior now. Each statement was rated on a 5-point Likert scale (0 = I strongly Disagree, to 4 = I strongly agree). Responses were summed to create a total score. The internal consistency of this scale was α = 0.82. The full scale is provided in the supplemental material of this article.

#### Reintegration

In consult with the study sample during the focus group discussions, we identified 8 items closely related to one’s readiness to reintegrate, the items were in line with the literature on the pull and push factors behind joining radical and terrorist groups [26, 32, 33, 71], some of the items were situated in the social context of joining, and others in the economic context. The instrument was created in Arabic, the first draft was then reviewed by a native Arabic-speaking psychologist who made necessary changes and modified the language accordingly, and another native Arabic psychologist reviewed the language and provided inputs, both versions were then compiled by the first author and changes were integrated. A group interview was held by the first author to test the language accessibility and the overall feasibility of the instrument with 15 participants of the study sample. Participants were asked to think about returning to their communities while answering the statements. Four items on the instrument represented positive expectations such as, “I expect to be able to protect myself—  ”, while the other four items represented negative expectations such as, “I fear to be marginalized—  ”. Each item was rated on a 5-point Likert scale, ranging from 0 = strongly disagree to 4 = Strongly agree. A. Negatively worded items were reverse coded, and a total score was calculated, where higher scores reflected a greater readiness for reintegration. The overall reliability of the scale was *α* = 0.86. The full scale is provided in the supplemental material of this article.

#### PTSD

Participants were asked to determine which of the events they mentioned so far is the worst event they have ever experienced, some specific questions from the life events checklist of the PTSD Checklist (PCL-5) were used to understand the worst event in more detail. To measure PTSD, the PTSD Checklist for DSM-5 [9] was used. PCL-5 is 20 questions scale that measures PTSD symptom severity across the four clusters of PTSD based on the DSM-5. Participants rated how much each symptom had bothered them in the past month, on a 5-point Likert Scale (0 = not at all, 4 = extremely) We used the validated Arabic translation of the instrument that was already used in Iraq [36]. The reliability of the scale was α = 0.86.

#### Depression

To assess depression, we used the Arabic translation of the Hopkins Symptoms Checklist for Depression [31] which is validated for use in Iraq. This checklist comprises 15 questions based on the DSM-5. Participants rated how much each symptom had bothered them in the last two weeks, then they rated each item using a 4-point Likert Scale ranging from 1 = not at all to 4 = extremely [36]. The scale’s reliability in the present study was α = 0.86. For an overview of all used instruments, please refer to Table 2.

**Table 2:** Overview of Instruments

measures	Number of items	Validation	Languages	References
Family violence	8	No	English and Arabic	[15, 16]
ISIS events	8	No	English and Arabic	Made by the authors of this paper
War Events	29	Yes	English and Arabic	[36]
Perpetration events	8	No	English and Arabic	Made by the authors of this paper
Identification with ISIS	17	No	English and Arabic	Made by the authors of this paper
Reintegration	8	No	English and Arabic	Made by the authors of this paper
PTSD	20	Yes	English and Arabic	[36]
Depression	15	Yes	English and Arabic	[36]

### Statistical analysis

To capture trauma exposure, we grouped all the scales that assessed trauma exposure, namely family violence, ISIS specific events, and war events in one composite variable that we named trauma exposure. In order to examine the relationship among the variables; trauma exposure, PTSD, depression, identification, and expectations for reintegration, a correlation analysis was conducted.

Bootstrapping was used to test whether depression mediated the relationship between trauma exposure and expectations for reintegration. Bootstrapped 95% Confidence Intervals (CIs) were generated using 1000 re-sampled datasets. This procedure entails re-sampling with replacement to generate a distribution of a given mediation effect. Bootstrapping is more powerful than conventional tests, such as Sobel’s z, because it considers the positive skew inherent to mediation effects [59]. Bootstrapping was conducted using Hayes’ (2022) PROCESS macro version 4 for SPSS [28], which generates bias-corrected CIs to further offset the aforementioned positive skew. All analyses were conducted using SPSS v. 28.

## Results

### War events

Our participants reported experiencing between 2 (1.7%) and 22 (3.4%) war events (M = 13.36, SD = 5.23). Thirteen events (11.9%) and 18 (11.9) were the most experienced events by the sample. Some of the most experienced events included imprisonment (94.9%), being tortured (86,4%), witnessing the destruction of residential areas (79.7%) having lost someone due to war being (76.3%), and witnessing dead bodies (74.6%).

### Family violence

Participants experienced a mean number of 2.84 different types of family violence events (SD = 2.10, range = 0–8). Being hit (81.4%), witnessing family members being hit (50.8%), and being told you are a bad son (50.8%) were the most experienced events.

### Mental health prevalence

Overall, 69.5% of the sample meet the criteria for a probable PTSD diagnosis using 33 as the standard, international cut-off score suggested for the instrument [9]. A total of 89.8% of the sample meet the criteria for a probable depression diagnosis.

### Depression as a mediator between expectations for reintegration and trauma exposure

**Fig. 1:**
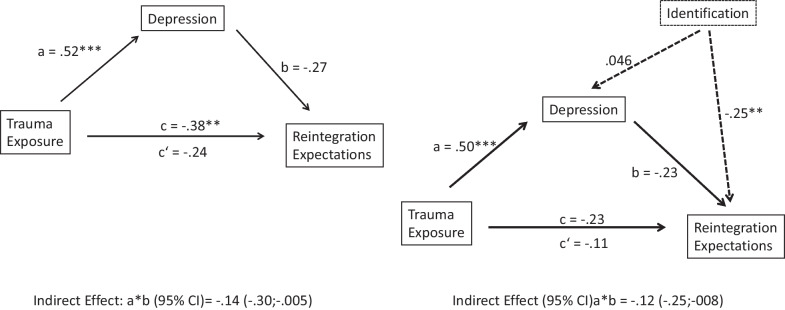
Mediation role of depression in the correlation between trauma exposure and expectations for reintegration, with and without controlling for identification with the armed group

The composite score for trauma exposure was negatively correlated with reintegration (β = − 0.88, *t* = 1.86 p < 0.05 and positively correlated with depression (β = 0.12, *t* = 4.08, *p* < 0.001). To examine the mediating role of depression in the association between trauma exposure and reintegration we tested a simple mediation model. Controlling for the mediating influence of identification with the terrorist group, the direct effect of multiple victimization events on expectation for reintegration reduced but was not significant (β = − 0.42, *t* = -0.80, *p* = 0.42) and when controlling for the mediating influence of identification with terrorist group, the direct effect of multiple victimization events on reintegration reduced but was not significant (β = − 0.42, *t* =− 0.80, *p* = 0.43). The indirect effect was tested with a bootstrap estimation procedure. The indirect effect of multiple victimization events predicting reintegration via depression reached significance (β = 0.46) 95% CI [− 1.0033, − 0.0284], see Fig. 1 (Table 3).

**Table 3:** Correlation between mental health, trauma exposure, perpetration, and reintegration

Variables	I	II	III	IV	V	VI	VII	VIII
Reintegration I	–		− .440^**^	− .376^**^	− .356^**^	− .463^**^	− .330*	− .384^**^
War events II		–	.884^**^	.737^**^	.446^**^	.357^**^	.607^**^	.489^**^
Trauma exposure III			–	.860^**^	.601^**^	.387^**^	.630^**^	.522^**^
ISIS events IV				–	.771^**^	.351^**^	.597^**^	.562^**^
Perpetration events V					–	.351^**^	.303^*^	.294^*^
Identification VI						–	.192	.241
PTSD VII							–	.765^**^
Depression VIII								–

^*^Correlation is significant at the 0.05 level (2-tailed)

^**^Correlation is significant at the 0.01 level (2-tailed)

## Discussion

Using clinical interviews with adolescents and young adults who were imprisoned in Iraq because of terrorism-related crimes we found that the individual’s positive expectations for reintegration was compromised by the ongoing identification with the terrorist organization but also trauma exposure and impaired mental health. In detail, we found that depression partially mediated the relationship between trauma exposure and expectation for reintegration even when the effects of identification were controlled for.

These findings reflect the recent history of war and unrest in Iraq with the vast majority (79.7%) having at least one event of displacement, and individuals reporting between 2 and 22 war events. This history of trauma is associated with high levels of psychopathology and, in turn, limits the expectations for successful reintegration.

Displacement is linked to many adverse life circumstances including poor mental health outcome [48, 68]. Children survivors of war suffer from immediate consequences such as increased domestic violence [51, 65] and intimate partner violence [25]. It is thought that children who witness violence as a norm to overcome challenges are at a greater risk of being violent themselves [13] and to make poor life choices [3].

Our findings suggest a strong relationship between war events and family violence, this replicates the already established literature on this [14–16, 19, 65]. War events was also strongly correlated with self-reported identification with the armed group and mental health. The relationship between war events and identification with armed groups can be understood through the lenses of social identity when both socialization into systems of violence and the feeling that ones’ social group is under attack can contribute to identifying with armed groups [69]. We also found that history of victimization (war events, family violence and ISIS specific events) was strongly correlated with perpetration events reported by the sample. This interaction could be possibly explained through the habituation to violence [42, 63] and the vicious cycle of violence [55].

Interestingly, the history of victimization and identification with the armed group is negatively correlated with expectations for reintegration in our sample but not self-reported perpetration events.

The high rates of PTSD and depression in our sample is elevated compared to previous studies in the literature, one explanation could be that the symptoms are exacerbated due to the juvenile detention [57], it could also be attributed to the unique experiences of our sample, having experienced war and conflict in the most formative years of their lives, having participated in armed groups and having been detained Our finding is consistent with prior research attesting to the deleterious impact of both war [50, 51, 70], and juvenile detention [80] on the mental wellbeing of young people. In the mediation analysis to find the effects of depression on the relationship between trauma exposure and readiness for reintegration, we found that depression only partly mediated the relationship between the two variables, suggesting that exposure to trauma has a very strong impact on readiness for reintegration. This mediation role could be explained by the negative thinking that defines depression. Compared to non-depressed individuals, depressed patients show an overall pattern of negative thinking [47, 64]. It is not only that patients with depression have negative thinking but studies also show that depressed patients show fewer positive anticipated experiences compared to non-depressed patients [45]. In a meta-analysis by [23], they found that people with depression struggle to imagine specific positive futures, this may explain the negative outlook our sample has on their prospects for reintegration.

The present study contributes to a small but growing, literature on the experiences of child soldiers by replicating and extending previous findings. In the understudied sample of children incarcerated for terrorism-related crimes, our findings suggest for peace to be a possibility, and for the vicious cycle of conflict and war to be ended, mental health can be the best approach. Our findings indicate that treating the effects of trauma and addressing identity and social identity are critically important targets for rehabilitation and reintegration programs for child soldiers or children involved in armed groups. However, studying such a sample does not come without its own compromises and limitations that must be considered when interpreting the results. First and foremost is the sample itself; even though every possible effort was made by the lead researcher to ensure the sample met the inclusion criteria, it is unclear if all participants would really qualify as child soldiers/children involved with armed groups or whether some individuals were falsely accused. In the interviews, the majority of participants admitted that they had joined ISIS, but the nature and number of perpetration events admitted by the participants were ostensibly low given the reasons for participants’ detainment, this could be attributed to the possibility of perpetration events being under-reported by the sample. Since we relied on interviewees being incarcerated for terrorism-related crimes, we were entirely dependent on the rule of law in Iraq, it is not inconceivable that some of the participants were indeed never a part of ISIS despite being convicted for the crimes. And that, 22% of the participants (13 respondents), denied having ever joined ISIS or any other terrorist group, 6 respondents went on and answered the identification with ISIS questions afterward, which might compromise the validity of the results. Another limitation is that the sample was self-selected, and thus not representative of former child soldiers/children involved with armed groups in this context or of all children within the juvenile facility itself. It is also important to note that this is not a causal study but rather a cross-sectional one, and as such the findings cannot give insights into the causes of terrorism but rather explains what the sample reported at a given point in time. The setting of the prison may have resulted in response biases. For instance, demand characteristics may have influenced interviewees to answer questions in a more socially desirable way, such as underreporting their agreement with radical ideas or perpetration of violence. Despite these limitations, the present study provides rare insights into the experiences of these individuals, and functions as an important step in advancing our understanding not just of the events that may predict reintegration, but also of the relationship between these factors. With the high prevalence of trauma related disorders, a culture of trauma informed practice might be the best way to bring about successful reintegration into the civil life for incarcerated youth who were recruited by armed groups. This study further emphasizes the critical role of mental health professionals in such regions and argues for special attention to supporting efforts towards strengthening local capacity in both academic institutions and on policymakers’ level in such countries.

## Conclusion

Despite the caution that should be practiced in interpreting the findings of the present study, our findings suggest that mental health, particularly depression, plays a vital role in the process of rehabilitation and reintegration of child soldiers. Accordingly, special consideration should be given to the processes of identity formation, especially in groups with a history of violence and oppression in the face of future reinvolvement with armed groups. In order to achieve a successful reintegration, trauma-informed interventions, tailored to the context of Iraq are essential. Furthermore, based on our findings and the overwhelming data in the literature [1, 80] on the negative impact of juvenile detention, it is likely that juvenile detention is not the best solution to rehabilitate child soldiers in the Iraqi context. Based on our sample's high prevalence of mental ill-health, we recommend an alternative approach. An approach that adapts trauma-informed work in the juvenile justice system in Iraq, where safety is provided for healing to occur before reintegration can happen. Having more longitudinal studies is crucial to understand reintegration in the context of Iraq better, it is equally important to consider mixed methods in addressing reintegration to understand the underpinning factors better, employing ethnographic, social-ecological, and anthropological studies will help understand pathways to reintegration better.

## Data Availability

The datasets used and/or analysed during the current study are available from the corresponding author on reasonable request.
